# Enhancing *Alcelaphine gammaherpesvirus 1* propagation in the MDBK cell line: role of ruxolitinib and reduced incubation temperature

**DOI:** 10.1007/s11259-026-11192-6

**Published:** 2026-04-01

**Authors:** Hana Malenovská

**Affiliations:** Collection of Animal Pathogenic Microorganisms, Veterinary Research Institute, Hudcova 70, 621 00 Brno, Czech Republic

**Keywords:** *Alcelaphine gammaherpesvirus 1*, MDBK, Ruxolitinib, Virus propagation

## Abstract

*Alcelaphine gammaherpesvirus 1* (AlHV-1) is the causative agent of malignant catarrhal fever, a fatal disease of cattle prevalent in regions with wildebeest populations. In vitro isolation and propagation of AlHV-1 is laborious and often requires multiple passages to improve the visibility of the cytopathic effect in host cells and increase virus yields. Ruxolitinib increases the replication of some viruses due to inhibition of the IFN-induced antiviral response of infected cells. In this study, the effect of a ruxolitinib-enriched culture medium was tested simultaneously with the effect of a reduced incubation temperature (33 °C) on the propagation of the AlHV-1 strain WC-11 adapted to the MDBK cell line. Ruxolitinib significantly increased the destruction of the cell monolayer during AIHV-1 propagation and significantly increased the virus titre. In addition, the amount of cell-free AIHV-1 particles in the culture medium was enhanced significantly in the presence of ruxolitinib. A reduced incubation temperature (33 °C), either in combination with ruxolitinib-enriched medium or without ruxolitinib, had no significant effect on AIHV-1 growth. These results demonstrate that ruxolitinib enhances the propagation of AlHV-1 strain WC-11 and suggest a broader potential to improve the efficiency of AlHV-1 cell culture-based applications, such as virus isolation, vaccine production, and diagnostics.

## Introduction

*Alcelaphine gammaherpesvirus 1* (AlHV-1) is a member of the genus *Macavirus* and the subfamily *Gammaherpesvirinae*. Together with *Ovine gammaherpesvirus 2*, it is the main causative agent of malignant catarrhal fever, a sporadic but fatal generalised disease in various ruminants, including domestic cattle. The natural reservoir for AIHV-1 is wildebeest, in which the infection is inapparent. The disease, which is associated with significant economic losses, occurs mainly in regions of Africa where the virus can be transmitted from wildebeest to cattle and other ruminants, or in zoological gardens worldwide (Russell et al. 2009).

The cultivation of AlHV-1 is a difficult procedure, as AlHV-1 is cell-associated. The cytopathic effect (CPE) during the cultivation of AlHV-1 in a cell monolayer can take many days to become visible, is usually focal and is followed by regrowth of the monolayer. In addition, AlHV-1 can be isolated primarily from cell suspensions of affected tissues due to its cell-association (WOAH 2018). However, AlHV-1 has been successfully propagated in some bovine cell cultures (Ferris et al. 1976; Horner and Tham 2003; Dewals and Vanderplasschen 2011; Hristov and Peshev 2016; WOAH 2018; WOAH 2020). Interestingly, Harkness and Jessett (1981) achieved increased AIHV-1 growth in bovine thyroid cells due to a reduced incubation temperature.

The use of ruxolitinib as an additive in the culture medium has been shown to increase the in vitro growth of some viruses, e.g. measles, hepatitis C and influenza A virus (Stewart et al. 2014; Cataldi et al. 2015; Wang et al. 2018; Malenovská 2023). Ruxolitinib is originally used for the treatment of myelofibrosis, polycythemia vera and steroid-refractory graft-versus-host disease and affects in vitro virus growth due to its inhibition of the IFN-induced antiviral response of infected cells through JAK inhibition (Appeldoorn et al. 2023). Therefore, this study examined the effects of ruxolitinib supplementation of the culture medium and a reduced incubation temperature on AlHV-1 growth.

## Materials and methods

### Virus strain and cell line

The AlHV-1 virus strain WC 11 used in this study originates from the Collection of Animal Pathogenic Microorganisms (CAPM) included in the National Programme for the Conservation and Utilisation of Microbial Genetic Resources and Invertebrates of Agricultural Importance in the Czech Republic. The virus was initially propagated in primary bovine kidney cells; for experimental propagation, the MDBK (Madin Darby bovine kidney) cell line (ATCC CCL-22) was used.

### Experimental design

MDBK cell cultures were grown in 25 cm^2^ flasks in Dulbecco’s Modified Eagle’s Medium (DMEM Low Glucose, Biosera, France) with 10% FBS (Serana Europe GmbH, Germany). One-day monolayer cultures were washed twice with phosphate-buffered saline (PBS) prior to virus inoculation. Each culture was inoculated with AlHV-1 at a multiplicity of infection (MOI) of 0.2, maintained at 37 °C for one hour to adsorb the virus and then DMEM with 3% FBS was added with or without ruxolitinib. Five independent flasks with cultures were subjected to the same combination of incubation temperature and medium composition. The combinations were as follows: (1) reduced incubation temperature (33 °C) and culture medium with addition of 4 µM ruxolitinib (InvivoGen, USA); (2) standard incubation temperature (37 °C), medium with addition of 4 µM ruxolitinib; (3) reduced incubation temperature, without ruxolitinib; (4) standard incubation temperature, without ruxolitinib. The flasks were incubated in a 5% CO_2_ atmosphere and frozen at -80 °C eight days post-infection (dpi). Three consecutive virus passages were performed under identical conditions prior to analysis. After the third passage, the CPE in the MDBK cell line was assessed at eight dpi under the light microscope as follows: (1) generalised cell destruction or (2) focal degeneration of the monolayer (American Society for Microbiology 2016). Aliquots of culture media were collected for quantification of cell-free virus by transmission electron microscopy (TEM); the flasks were then frozen at -80 °C for subsequent TCID_50_ determination.

### TCID_50_ assay

For the TCID_50_ assay, the AlHV-1 suspension was rapidly frozen (-80 °C) and thawed three times (37 °C water bath) to disrupt the cells, and serial 10-fold dilutions were prepared using culture medium with 3% FBS as diluent. A 48-well microplate with a confluent monolayer of MDBK was inoculated with virus dilutions in a similar manner as previously described (Malenovská 2023). The culture medium containing 3% FBS was added after infection, and the plates were incubated for 8 days at 37 °C in 5% CO_2_ atmosphere before being analysed by light microscopy and *lg* TCID_50_ quantified according to Kaerber (1931).

### Transmission electron microscopic (TEM) quantification of the virus

A modification of a previously described TEM-based virus quantification method (Malenovská 2013) was applied to compare the relative abundance of cell-free AlHV-1 particles in the culture medium in different suspensions. Briefly, the suspensions were centrifuged at 1500 x g for 10 min to remove cell debris and diluted 1:4 with distilled water. A 400-mesh copper grid coated with Formvar carbon was placed on a 30 µl drop of the suspension and allowed to adsorb for 1 min. The grids were stained with 1.5% ammonium molybdate for 1 min and viewed in a Philips EM 208 transmission electron microscope at 11,000× magnification. The number of virus particles in 10 random grids was counted to determine the average number of particles for a grid.

### Statistical analysis

The effect of adding ruxolitinib to a culture medium and lowering the incubation temperature on the probability of development of generalised CPE in the MDBK cell line was tested using a generalised linear model with an assumed binomial distribution (GLM-b, Pekár and Brabec 2016). The significance of the influence of the factors on the CPE was quantified by χ^2^ statistics. The final model without insignificant variables was used to estimate the probability of development of generalised CPE under specific conditions. The 95% confidence intervals were calculated to assess the accuracy of the model. A two-way ANOVA test was used to evaluate the effects of the addition of ruxolitinib to the culture medium and reduced incubation temperature on virus production (TCID_50_ or TEM particle count). Statistical tests were considered significant at *P* < 0.05 and highly significant at *P* ˂ 0.001. All analyses were performed using R Statistical Software version 4.4.2 (R Core Team 2024).

## Results

**Fig. 1:**
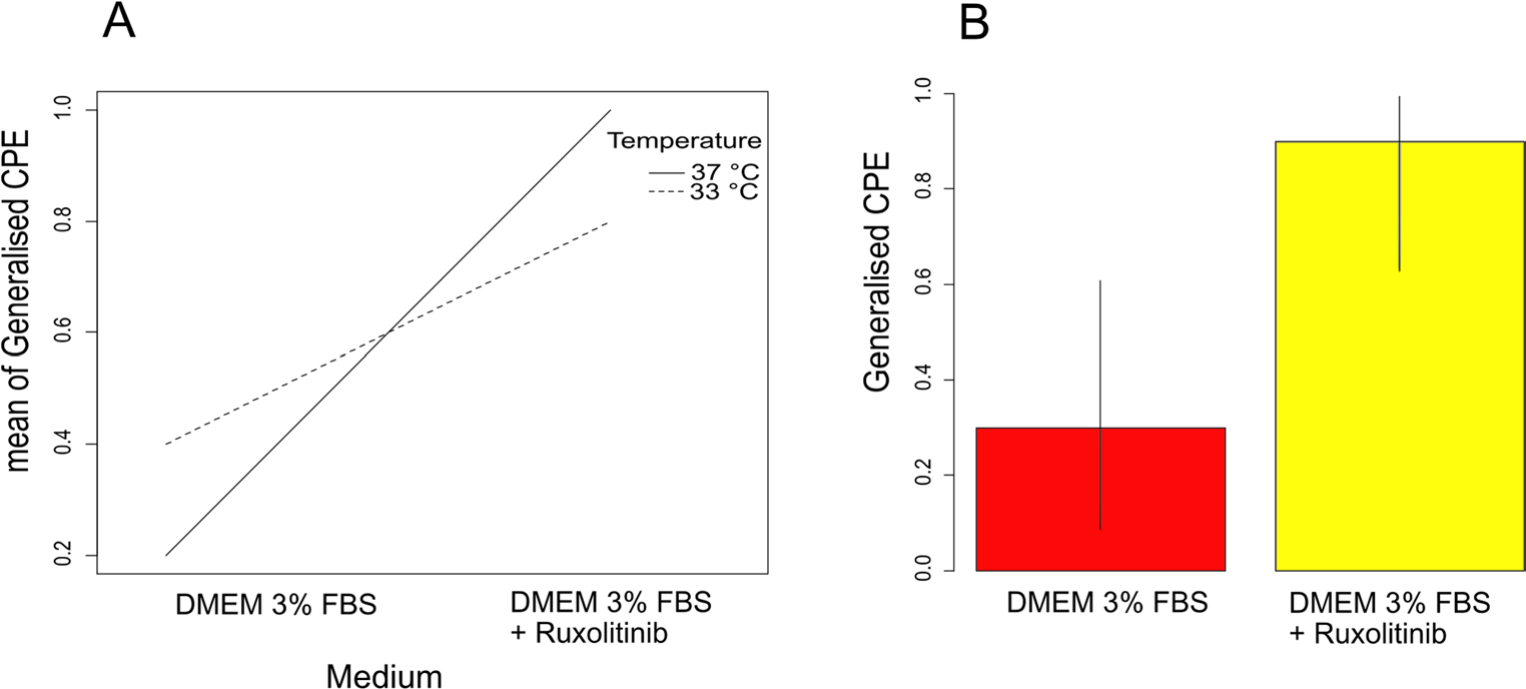
Development of CPE characterised by a generalised destruction of MDBK cells during AIHV-1 growth under different medium compositions and incubation temperatures: **A** Comparison of mean values for the development of generalised CPE. **B** Estimates of the development of generalised CPE based on GLM-b. The vertical lines show 95% confidence intervals

The results of the CPE evaluation in AlHV-1-infected MDBK cells under different medium compositions and incubation temperatures are shown in Fig. 1A. In medium with ruxolitinib, progression of CPE up to generalised destruction of the cell monolayer was achieved in four out of five cases at 33 °C and in all five cases at 37 °C. In medium without ruxolitinib, the frequency of development of generalised CPE was lower, two at 33 °C and one in five at 37 °C. The probability of the development of the generalised CPE was significantly influenced by the addition of ruxolitinib in the medium (GLM-b, χ^2^_1_ = 8.2, *P* = 0.004). The influence of incubation temperature was not significant (*P* = 1). The effect of ruxolitinib was the same at both temperatures, as the interaction between ruxolitinib addition and incubation temperature was not significant (*P* = 0.16). The probability of the development of generalised CPE in the AlHV-1 infected cells, which was influenced by the addition of ruxolitinib to the culture medium, is shown in Fig. 1B.

**Fig. 2:**
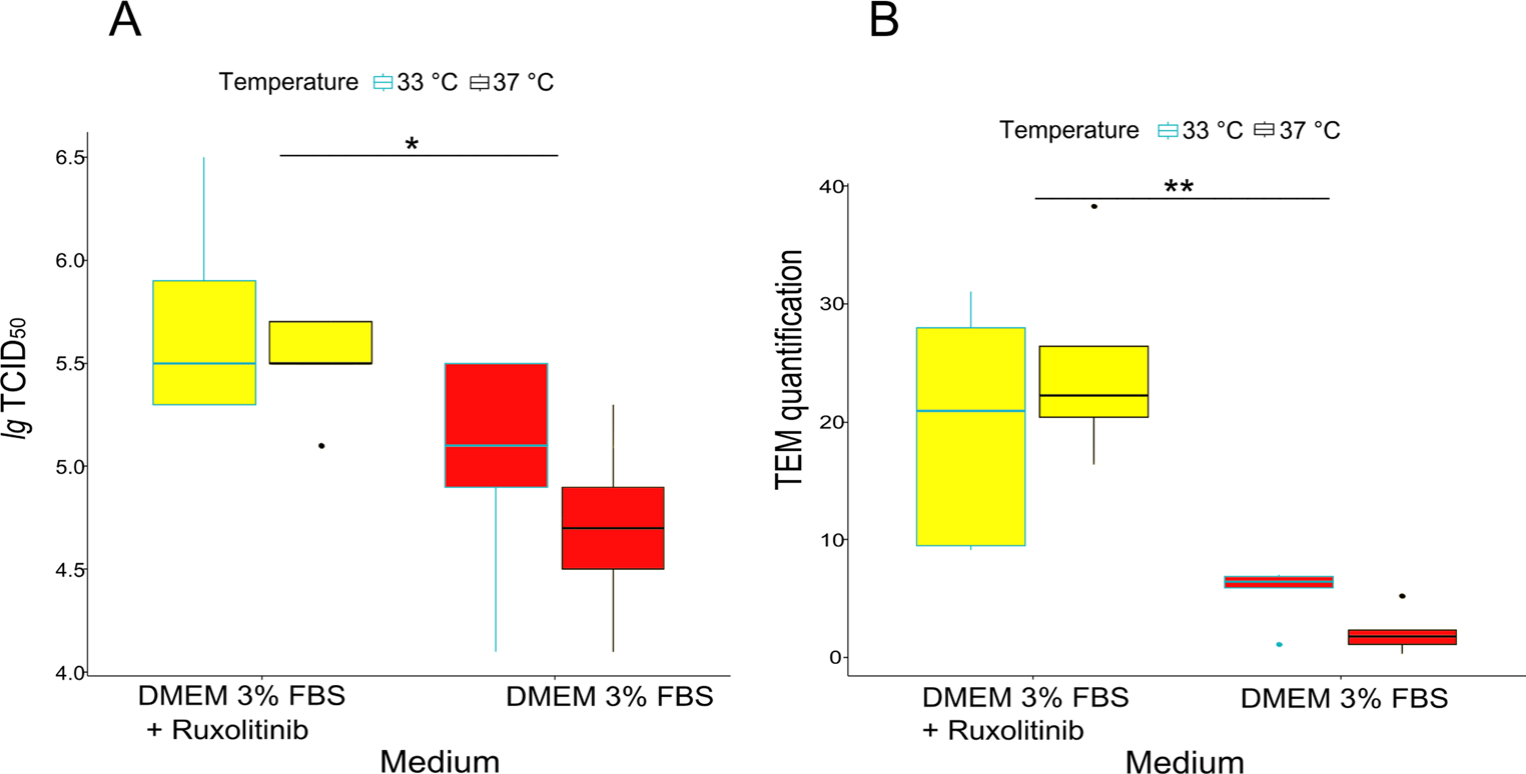
Yields of AlHV-1 in the MDBK cell line under different culture conditions: **A** Infectious titres (*lg* TCID_50_). Differences between titres were considered significant (*) at *P* ˂ 0.05. **B** Results of TEM quantification of cell-free AlHV-1 particles. Differences between virus titres were considered highly significant (**) at *P* ˂ 0.001

The effects of the addition of ruxolitinib to the culture medium at different incubation temperatures on the virus titres (*lg* TCID_50_) are shown in Fig. 2A. The addition of ruxolitinib to the culture medium promoted AlHV-1 growth, resulting in a significant increase in AlHV-1 titres (*P* = 0.0025). The effect of incubation temperature was not significant (*P* = 0.23) and the effect of ruxolitinib was independent of temperature, as the interaction between the two factors was not significant (*P* = 0.78). The influence of the tested culture conditions on the amount of cell-free AlHV-1 particles measured by TEM is shown in Fig. 2B. Ruxolitinib increased the amount of cell-free virus particles in the culture medium highly significantly (*P* = 0.00002). The change in incubation temperature was not significant (*P* = 0.78), nor was the interaction between ruxolitinib addition and incubation temperature (*P* = 0.19).

## Discussion

In this study, the addition of ruxolitinib to the culture medium increased the probability of developing generalised CPE at the third passage in MDBK threefold compared to a culture without ruxolitinib (Fig. 1). This was accompanied by a substantial increase in both the amount of cell-free AlHV-1 particles and the overall infectious titres (*lg* TCID_50_) of AlHV-1 (Fig. 2), consistent with increased viral replication. After repeated passages of AlHV-1, the character of CPE in cell culture changes from a focal to a generalised destruction of the cell monolayer (Ferris et al. 1976), which is accompanied by an increased proportion of cell-free viruses that ensure increased infection of the remaining cells (Russell et al. 2009; Hristov and Peshev 2016; WOAH 2018). According to the result of this study, the whole process can be facilitated by the addition of ruxolitinib to the culture medium, which causes inhibition of the IFN-induced antiviral response of host cells (Appeldoorn et al. 2023). Although only certain herpesviruses and variants of herpesvirus species are significantly affected by IFN (Poelaert et al. 2018), AlHV-1 appears to be IFN-sensitive, also in agreement with Wan et al. (1988).

In contrast to Harkness and Jassett (1981), a reduced incubation temperature had no significant effect on the AlHV-1 titre or the character of the CPE. However, the different results of the studies were obtained in different host cells and thus indicate that the temperature-dependent AlHV-1 growth is related to temperature-dependent properties of certain cells, e.g. gene expression.

In summary, supplementation of the culture medium with ruxolitinib promotes the growth of the attenuated laboratory AlHV-1 strain WC 11 in the MDBK cell line and may offer significant advantages for AlHV-1 propagation and isolation in general.

## Data Availability

The data supporting the findings of this study are included in the published article. Additional information is available from the corresponding author upon reasonable request.
